# Comparison of Clinical Features and Outcomes of Medically Attended COVID-19 and Influenza Patients in a Defined Population in the 2020 Respiratory Virus Season

**DOI:** 10.3389/fpubh.2021.587425

**Published:** 2021-03-23

**Authors:** Long Liu, Feng Zeng, Jingjing Rao, Shengren Yuan, Manshan Ji, Xu Lei, Xiao Xiao, Zhijun Li, Xiaohua Li, Weixing Du, Yanqing Liu, Huabing Tan, Junmin Li, Jianyong Zhu, Jing Yang, Zhixin Liu

**Affiliations:** ^1^Department of Infectious Diseases, Renmin Hospital, School of Basic Medical Sciences, Hubei University of Medicine, Shiyan, China; ^2^Department of Respiratory, Renmin Hospital, School of Basic Medical Sciences, Hubei University of Medicine, Shiyan, China; ^3^Hubei Key Laboratory of Embryonic Stem Cell Research, Hubei University of Medicine, Shiyan, China; ^4^Department of Emergency, Dongfeng Maojian Hospital, Sinopharm Group Corporation, Shiyan, China

**Keywords:** influenza, COVID-19, epidemic, comparison, adaptive mutation

## Abstract

The emergence of severe acute respiratory syndrome coronavirus 2 (SARS-COV-2), which is causing the coronavirus disease-2019 (COVID-19) pandemic, poses a global health threat. However, it is easy to confuse COVID-19 with seasonal influenza in preliminary clinical diagnosis. In this study, the differences between influenza and COVID-19 in epidemiological features, clinical manifestations, comorbidities and pathogen biology were comprehensively compared and analyzed. SARS-CoV-2 causes a higher proportion of pneumonia (90.67 vs. 17.07%) and acute respiratory distress syndrome (12.00 vs. 0%) than influenza A virus. The proportion of leukopenia for influenza patients was 31.71% compared with 12.00% for COVID-19 patients (*P* = 0.0096). The creatinine and creatine kinase were significantly elevated when there were COVID-19 patients. The basic reproductive number (R_0_) for SARS-CoV-2 is 2.38 compared with 1.28 for seasonal influenza A virus. The mutation rate of SARS-CoV-2 ranges from 1.12 × 10^−3^ to 6.25 × 10^−3^, while seasonal influenza virus has a lower evolutionary rate (0.60-2.00 × 10^−6^). Overall, this study compared the clinical features and outcomes of medically attended COVID-19 and influenza patients. In addition, the S477N and N439K mutations on spike may affect the affinity with receptor ACE2. This study will contribute to COVID-19 control and epidemic surveillance in the future.

## Introduction

An increasing number of COVID-19 cases have caused a global health burden due to the rapid transmission throughout the human community. The pathogen SARS-COV-2 causes respiratory system and severe systemic symptoms through respiratory tract infection (1, 2). As of February 17th, 2021, more than 100 million COVID-19 cases were confirmed, and more than 2 million deaths had occurred (3). Although many countries are developing vaccines and racing to run clinical trials (4), there are still many unresolved questions regarding viral invasion, pathogenesis and clinical features. In particular, the relationship between mutations and pathogenicity or transmissibility remains unknown.

The seasonal influenza epidemic is caused by strains of influenza virus, including two influenza A viruses (H1N1 and H3N2) and one influenza B virus. There have been four documented influenza pandemics in the past 100 years (in 1918, 1957, 1968, and 2009) (5). The influenza case fatality rates of the 1918 and 2009 H1N1 pandemics ranged from 0.1% to 2.5% (6, 7). The very important issue is the emergence of a new subtype or strain through uncontrollable and unpredictable mutations or antigenic drift and shift (8). The same situation may also occur with SARS-COV-2. Some studies have reported spike mutations and attempted to clarify the transmissibility change associated with new mutations (9, 10).

Currently, SARS-COV-2 remains a lasting threat to public health, causing mild respiratory system disease similar to that caused by seasonal influenza virus. To investigate the COVID-19 clinical progression, prognosis and SARS-COV-2 epidemic trends, we systematically contrasted the proportions or values of epidemiologic characteristics, clinical features, blood abnormalities, progressive symptoms, and hospitalization rates between influenza and COVID-19. Seasonal influenza was prevalent in winter, and COVID-19 also emerged in the last winter. The parameters of coinfection patients who presented with both COVID-19 and flu were also compared with those of COVID-19 patients. In addition, the mutants of influenza virus HA and SARS-COV-2 spike causing the epidemic were resolved and discussed. We expect that this comparative research will aid pandemic control and be beneficial to the clinical diagnosis of SARS-COV-2.

## Materials and Methods

### Ethics

This study was approved by the Institutional Review Committee of Shiyan Renmin Hospital of Hubei University of Medicine, and no informed consent was required. This study was designed as a retrospective case analysis, with no patients directly involved in the study design, question setting, or outcome evaluation.

### Study Population

From January 23, 2020 to February 27, 2020, the confirmed COVID-19 patients and influenza patients on outpatient visits and admission to Shiyan Renmin Hospital were uniformly collected. The clinical data of 75 COVID-19 patients, 41 influenza patients and 23 coinfection patients were retrospectively collected at the same time. All patients underwent chest computed tomography (CT) scanning at admission. Flu patients were infected with the seasonal influenza A or B virus, COVID-19 patients were infected with SARS-COV-2, and coinfection patients were infected with both SARS-COV-2 and influenza A or B virus or parainfluenza virus (PIV). The clinical subtype for COVID-19 was screened according to the Diagnosis and Treatment Protocol for Novel Coronavirus Pneumonia (Trial Version 7) which was released by the National Health Commission & National Administration of Traditional Chinese Medicine (11). Influenza diagnosis and treatment plan (2019 version) (12) was used for screening the flu patients.

### Laboratory Examinations

After admission to the hospital, specimens from all patients were screened for SARS-COV-2 using throat swabs. The positive result of real-time reverse transcription-polymerase chain reaction (RT-PCR) confirmed the infection. Other respiratory pathogens were detected by indirect immunofluorescent assay using IgM antibodies, including *Legionella pneumophila, Coxiella burnetii, Chlamydia pneumoniae, Mycoplasma pneumoniae*, adenovirus (AdV), influenza A virus, influenza B virus, parainfluenza virus (PIV type 1+2+3), and respiratory syncytial virus (RSV). Sputum or body fluids were also examined at admission for other possible infections with bacteria or fungi.

### Data Collection

The epidemiological data, symptoms, laboratory abnormalities on admission, clinical treatments, and outcomes were recorded and collected. The examination of WBC, influenza virus antigen, joint test of nine respiratory tract pathogens, procalcitonin (PCT), hypersensitive C-reactive protein (CRP), erythrocyte sedimentation rate (ESR), blood gas analysis, and chest computed tomography (CT) were completed in the hospital. The above information was extracted from the case database of Shiyan Renmin Hospital.

### Sequence Alignment and Mutation Analysis

The genome sequences of SARS-COV-2 were downloaded from the GISAID database (13). Multiple sequences were aligned using MAGE-X software (version 10.0.5), phylogenetic analysis was completed through multiple comparisons using neighbor-joining algorithms, and the number of bootstraps was 500. All spike mutations were referenced to the website of the SARS-COV-2 Sequence Analysis pipeline (14).

### Dynamic Analysis of HA and Spike Structure

To analyze the dynamic model of the spike protein, we used the PDB file 6VXX. The model 1RUZ was used to validate the HA structure of influenza. PyMOL software (version 2.3.2) was used to map the S477N and N439K domain onto the 3D structure. MM/GBVI was used to calculate the binding free energy of each conformation with receptor ACE2 (15), ACE2 (PDB ID: 1R42) was used for computation.

### Statistical Analysis

SPSS 22.0 software (SPSS, Inc. Chicago, USA) was used for statistical analysis of the obtained data, and the measurement data are shown as the medians and interquartile ranges (IQRs), which were compared with the Mann-Whitney *U*-test. The categorical variables are shown as numbers (%) and were compared with the χ^2^ test or Fisher's exact test.

## Results

### Epidemiological and Population Characteristics of COVID-19 and Influenza Patients

From January 23, 2020 to February 27, 2020, a total of 141 suspected patients were admitted to the isolation ward of our hospital, of which 75 were diagnosed with COVID-19 pneumonia, and 23 patients were determined to be coinfected with influenza A or B or parainfluenza virus. As shown in Table 1, the median age of COVID-19 patients was 47.93 years old, and there were 42 males and 33 females, which included 66 mild patients and 9 critically ill patients. The male/female ratio of influenza patients was 1.05, while that of COVID-19 patients was 1.27. COVID-19 patients with SARS-COV-2 infection had a longer incubation period of 5.1 days, while flu patients developed symptoms after 1.4 days. Perhaps the longer incubation period of SARS-COV-2 is adverse to the epidemiological investigations, as 18–21% of COVID-19 patients did not have a clear infection path. There may have been more asymptomatic patients among the flu patients, as only 24% of patients could be traced to an infection source. COVID-19 patients had a severe rate of 12%, and coinfection patients had a higher severe proportion (21.74%).

**Table 1:** Demographic and epidemiologic characteristics of flu, COVID-19 and coinfection patients.

	**Flu (Influenza A/B,** ***n* = 41)**	**COVID-19** **(*n* = 75)**	**Coinfection (Influenza A/B, PIV *n* = 23)**
Age	48.21 ± 10.30	47.93 ± 10.55	54.75 ± 9.24
Gender ratio (M/F)	1.05	1.27	1.3
Incubation period (days)	1.4 (1.3, 1.5)	5.1 (4.5, 5.8)	4.9 (4.1, 5.9)
Severity of illness	3 (7.32%)	9 (12.00%)	5 (21.74%)
Clear epidemiology history	10 (24.39%)	59 (78.67%)	19 (82.61%)

### Clinical Characteristics and Laboratory Tests of Patients in Different Groups

The clinical characteristics of the patients are shown in Table 2. Some COVID-19 patients, flu patients and coinfection patients had the common manifestation of fever and cough. A total of 69.33 and 9.33% of COVID-19 patients had fever and nasal obstruction and rhinorrhea, respectively, which were all lower rates than those for the flu patients (*P* = 0.0263, *P* = 0.0083). Fatigue reported in 36% of COVID-19 patients was higher than the rate in flu patients (*P* = 0.0023). Patients coinfected with SARS-COV-2 and influenza virus or PIV had a lower cough rate than COVID-19 patients (*P* = 0.0310). Other symptoms included headache, nausea, vomiting, and diarrhea. For routine blood tests, a higher proportion of flu patients developed leukopenia than COVID-19 patients (31.71 vs. 12.00%, *P* = 0.0096), while coinfection patients had a decreased proportion (4.35%) of leukopenia relative to that of the COVID-19 patients, but there was no significant difference (*P* = 0.2889). A total of 70.67% of COVID-19 patients had increased CRP, sharing a median of 13.63 mg/L, which was higher than that of the flu group (*P* = 0.0372). The median of an increased proportion of ESR was not significantly different among the three groups. The increase of ESR (48.00 vs. 53.66%) and D-dimer (37.33 vs. 36.58%) was a common phenomenon in the COVID-19 and flu patients. The creatinine was significantly increased in the COVID-19 group than flu group (*P* = 0.0239), while creatine kinase was elevated when there were COVID-19 patients (16.00% or 30.43 vs. 2.44%).

**Table 2:** Clinical characteristics and selected laboratory abnormalities of flu, COVID-19 and coinfection patients.

	**Influenza (*n* = 41)**	**COVID-19 (*n* = 75)^**a**^**	**Coinfection (Influenza A/B, *n* = 23)^**b**^**	***P* value^**a**^^,^^**b**^**
**Clinical characteristics**				
Fever(≥ 37.3°C)	36 (87.80%)	52 (69.33%)	15 (65.22%)	0.0263, 0.7989
Cough	21 (51.22%)	49 (65.33%)	9 (39.13%)	0.1662, 0.0310
Nasal obstruction and rhinorrhea	12 (29.27%)	7 (9.33%)	5 (21.74%)	0.0083, 0.1454
Sore throat	4 (9.76%)	14 (18.67%)	4 (17.39%)	0.2855, 1.0000
Shortness of breath and chest tightness	3 (7.32%)	9 (12.00%)	2 (8.70%)	0.5353, 1.0000
Fatigue	4 (9.76%)	27 (36.00%)	6 (26.09%)	0.0023, 0.3788
Diarrhea and vomiting	3 (7.32%)	7 (9.33%)	2 (8.70%)	1.0000, 1.0000
**Blood routine**				
WBC count(× 10^9^/L)	5.18 (3.71, 8.12)	5.52 (4.19, 7.24)	5.34 (4.51, 6.23)	0.2112, 0.6358
(≤ 3.5 × 10^9^/L)	13 (31.71%)	9 (12.00%)	1 (4.35%)	0.0096, 0.2889
Lymphocyte count (× 10^9^/L)	1.20 (0.83, 1.62)	1.23 (0.90, 1.59)	1.39 (1.05, 1.76)	0.9678, 0.0627
(≤ 1.1 × 109/L)	15 (36.59%)	20 (26.67%)	5 (21.74%)	0.2659, 0.6353
CRP (mg/L)	7.43 (4.26, 17.40)	13.63 (3.93, 26.60)	14.82 (4.87, 28.41)	0.0617, 0.2026
(≥5mg/L)	21 (51.22%)	53 (70.67%)	14 (60.87%)	0.0372, 0.3768
ESR(mm/h)	18 (7.50, 33.50)	17 (7.00, 31.50)	21.5 (9.50, 41.00)	0.4271, 0.5078
(≥15 mm/h)	22 (53.66%)	36 (48.00%)	12 (52.17%)	0.6979, 0.7261
(D-dimer mg/L)	0.17 (0.10, 0.28)	0.25 (0.14, 0.34)	0.28 (0.18, 0.37)	0.0046, 0.1246
(≥0.25 mg/L)	15 (36.58%)	28 (37.33%)	12 (52.17%)	0.4311, 0.0018
Creatine kinase (U/L)	104 (66.30, 149.60)	118 (78.50, 158.30)	115 (62.40, 175.20)	0.1157, 0.8316
(≥171 U/L)	1 (2.44%)	12 (16%)	7 (30.43%)	0.0013, 0.0027
BUN (mmol/L)	4.43 (3.69, 5.26)	4.38 (3.52, 5.21)	4.50 (3.76, 5.03)	0.9550, 0.5377
Creatinine (μmol/L)	86.32 (78.90, 92.90)	90.94 (74.70, 108.10)	97.99 (76.70, 118.04)	0.0008, 0.1659
(≥104μmol/L)	3 (7.32%)	18 (24%)	6 (26.09%)	0.0239, 0.5840

*The data was shown as n (%) or median (IQR)*.

a *COVID-19 vs. Influenza group*.

b *Coinfection vs. COVID-19 group*.

### Radiological Finding and Clinical Outcome Comparison

**Figure 1:**
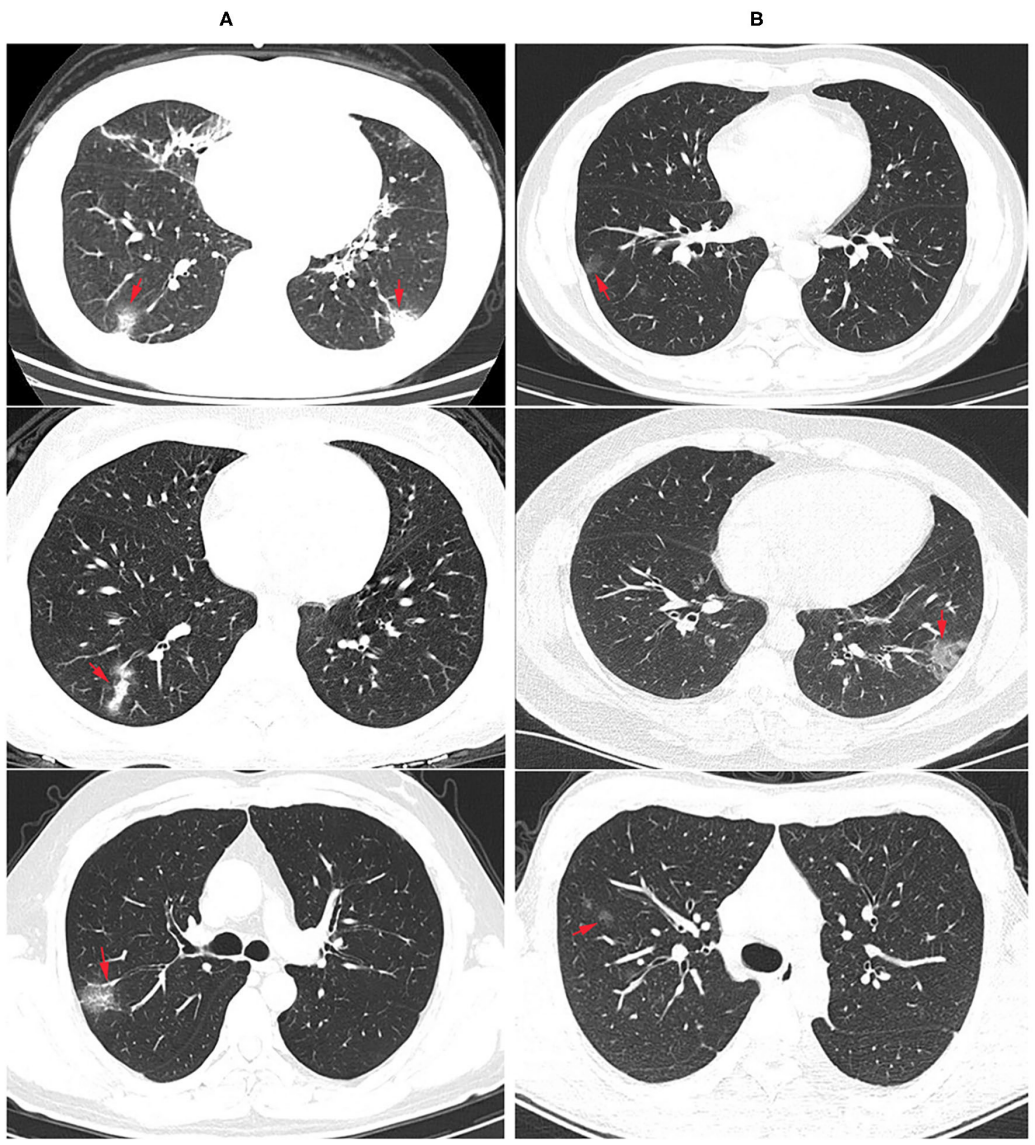
Radiological findings: chest computer tomography (CT) images of COVID-19 and flu pneumonia at the same time after onset. **(A)** COVID-19 pneumonia showed multiple ground glass opacity with solid components in the bilateral subpleural area. The nucleic acid test for SARS-CoV-2 was positive. Arrows showed the lesions. **(B)** CT examination showed scattered ground glass opacity of both lungs in an influenza patient, mainly in the lung periphery with less solid components. SARS-CoV-2 nucleic acid test was negative for three times, and immunofluorescence test was positive for influenza A virus.

Among the 75 confirmed COVID-19 patients, 45 showed typical signs of viral pneumonia on chest CT. The other 23 patients showed lung infections but no typical signs on chest CT. In total, 90.67% of COVID-19 patients developed pneumonia, which is much higher than the proportion of influenza patients with pneumonia (17.07%, *P* < 0.0001) (Table 3), and this proportion increased to 95.65% in coinfection patients. The lesions for COVID-19 pneumonia were mostly in the subpleural area, with patchy or lumpy appearance (Figure 1A). The density of the lesions was commonly ground-glass opacities (GGOs), and there were real changes and thickened leaflet intervals. Influenza pneumonia showed GGOs with fewer solid components (Figure 1B). Twenty-one COVID-19 patients (28%) were diagnosed with underlying diseases (Table 3), and the top three were hypertension (13.33%), diabetes (8%), and coronary heart disease (5.33%). The proportion and order of underlying diseases in the flu group and coinfection group were consistent. A total of 18.67% of COVID-19 patients developed complications of liver injury, which was a higher rate than that of flu patients (4.88%, *P* = 0.0395). No flu patient had a complication of acute respiratory distress syndrome (ARDS), with a proportion of 12% in the COVID-19 group (*P* = 0.0209). In the coinfection group, ARDS was the most common complication (17.39%), followed by liver injury (13.04%) and kidney injury (8.70%).

**Table 3:** Underlying diseases and progressive symptoms of flu, COVID-19 and coinfection patients.

	**Influenza (*n* = 41)**	**COVID-19 (*n* = 75)^**a**^**	**Coinfection (Influenza A/B, *n* = 23)^**b**^**	***P* value^**a**^^,^^**b**^**
Underlying diseases	11 (26.83%)	21 (28.00%)	7 (30.43%)	1.0000, 0.7981
Hypertension	4 (9.76%)	10 (13.33%)	3 (13.04%)	0.7675, 1.0000
Diabetes	3 (7.32%)	6 (8.00%)	2 (8.70%)	1.0000, 1.0000
Coronary heart disease	3 (7.32%)	4 (5.33%)	1 (4.35%)	0.6965, 1.0000
**Progressive symptoms**				
Pneumonia	7 (17.07%)	68 (90.67%)	22 (95.65%)	< 0.0001, 0.4449
Acute respiratory distress syndrome	0	9 (12.00%)	4 (17.39%)	0.0209, 0.5049
Shock	0	1 (1.33%)	0	1.0000, 1.0000
Liver injury	2 (4.88%)	14 (18.67%)	3 (13.04%)	0.0395, 0.5333
Kidney injury	0	6 (8.00%)	2 (8.70%)	0.0629, 0.9151

*The data was shown as n (%)*.

a *COVID-19 vs. Influenza group*.

b *Coinfection vs. COVID-19 group*.

### Hospitalization and Treatment for Patients in Different Groups

The median hospitalization period of COVID-19 patients was 19 days, while flu patients after admission required only 4 days to discharge (*P* < 0.0001) (Table 4). According to the Diagnosis and Treatment Protocol for COVID-19 (11), all COVID-19 patients received broad-spectrum antiviral treatment, including interferon-α sprays, arbidol hydrochloride, or lopinavir and ritonavir (16). Antibiotics, including cephalosporins, carbapenem, quinolones and so on, and antifungal drugs were used when appropriate. By the end of the study period, all patients were being treated in the hospital, and 98.67% of COVID-19 patients were cured and survived; only one patient died (1.33%). All flu and coinfection patients were cured and discharged.

**Table 4:** Hospitalization for flu, COVID-19 and coinfection patients.

	**Influenza (*n* = 41)**	**COVID-19 (*n* = 75)^**a**^**	**Coinfection (Influenza A/B, *n* = 23)^**b**^**	***P* value^**a**^^,^^**b**^**
Hospitalization period (days)	3.95 (3, 5)	19.12 (11, 26)	19.48 (13, 25)	< 0.0001, 0.5424
Treatment	Oseltamivir, Peramivir	Following the guideline	Oseltamivir, Peramivir (for influenza A/B)	
Cure rate	41 (100%)	74 (98.67%)	23 (100%)	0.4577, 0.5778
Fatality rate	0	1 (1.33%)	0	0.4577, 0.5778

*The data was shown as n (%)*.

a *COVID-19 vs. Influenza group*.

b *Coinfection vs. COVID-19 group*.

### Pathogen Comparisons for Influenza Virus, SARS-COV and SARS-COV-2

Influenza virus belongs to the family *Orthomyxoviridae*, whose genome contains eight RNA segments. SARS-COV and SARS-COV-2 belong to the β-coronavirus (CoV) genus in the *Coronaviridae* family (17). SARS-COV-2 showed a high nucleotide sequence identity (79.5%) with SARS-COV (18). Recent reports have shown that SARS-COV-2 enters susceptible cells through binding with the receptor angiotensin-converting enzyme 2 (ACE2), which is the same as SARS-COV (19–21). All ages of people are susceptible to influenza A virus (22), while SARS-COV and SARS-COV-2 primarily infect adults (23). Because all three viruses can bind with receptors in the upper respiratory tract of humans, they are all easily transmitted by airborne droplets during coughing, sneezing or intimate conversation. The reproductive number (R_0_) is defined as the average number of secondary cases generated per confirmed infectious case. The reported median R_0_ for seasonal influenza virus is 1.28 (IQR: 1.19–1.37), except for during the 2009 pandemic (Table 5) (24). SARS-COV-2 had a median R_0_ of 2.38 at the beginning of the epidemic (25), which is still controversial, but it is probably higher than the R_0_ of SARS-COV (1.7-1.9) (26). Surveillance of genome mutation dynamics is critical for the effective control of diseases. To date, SARS-COV-2 seems to exhibit a higher mutation rate than influenza virus per site per year [(1.12-6.25) × 10^−3^ vs. (0.60-2.00) × 10^−6^] (27–29), which remains to be further determined. Relatively, SARS-COV has a similar evolution rate (0.80-2.38 × 10^−3^ per site per year) (30).

**Table 5:** Pathogen comparisons for seasonal flu virus, SARS-COV-2 and SARS-COV.

	**Seasonal influenza virus (H1N1, H3N2)**	**SARS-COV-2**	**SARS-COV**
Family	*Orthomyxoviridae*	*Coronaviridae*	*Coronaviridae*
Susceptible crowd	Children and adults	Children and adults	Adults
Transmission	Droplets	Droplets	Droplets
R_0_	1.28 (IQR: 1.19–1.37)	2.38 [95% (CI): 2.03–2.77]	1.7-1.9
Mutation (/site/year)	0.60-2.00 × 10^−6^	1.12-6.25× 10^−3^	0.80-2.38 × 10^−3^

### Adaptive Mutations of Influenza Virus and SARS-COV-2

**Figure 2:**
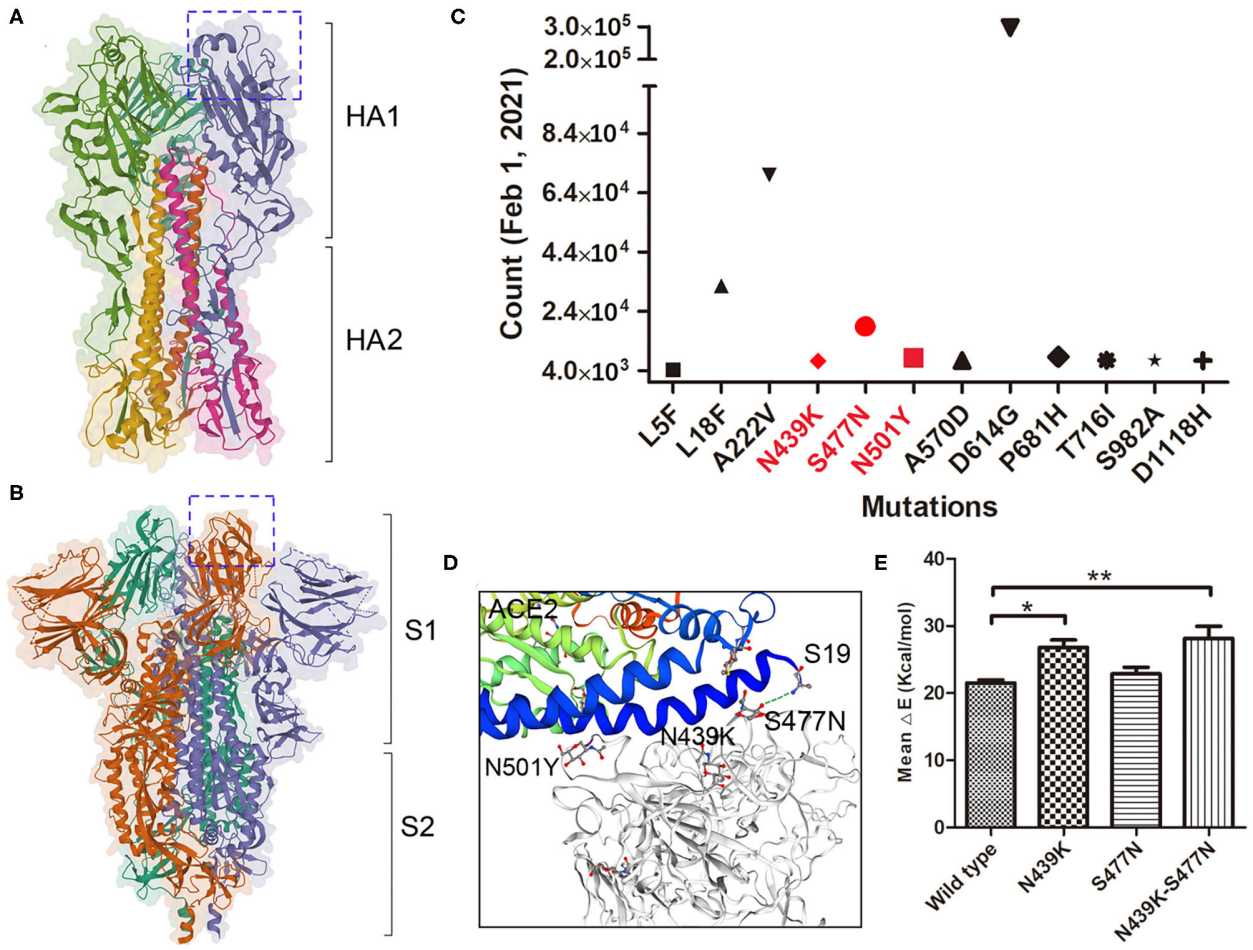
Mutations on SARS-COV-2 spike protein. **(A)** The trimeric structure of influenza viral HA, each monomer is composed with HA1 and HA2 domain. The receptor-binding pocket were marked in box. **(B)** The trimeric structure of SARS-COV-2 spike protein, each monomer is composed with S1 and S2 domain. The receptor-binding domain (closed) were marked in box. **(C)** The major mutations on spike protein up to February 1st, 2021. Mutations in Red are located within the RBD. **(D)** The mutations binding with the receptor ACE2. Stimulation shown the residue S477N binding with ACE2 Sernine19. **(E)** The binding affinity of wild type and variants of spike with ACE2 was calculated by MM/GBVI. Data are the mean ± SD, n = 3. *P < 0.05, **P < 0.01, compared with the wild type (one-way ANOVA and Tukey's *post hoc* test).

Haemagglutinin (HA) on influenza virus and spike on SARS-COV-2 are both responsible for binding to the receptors on permissive cells. HA and spike are both homotrimers, where each monomer comprising two subunits, HA1 and HA2 or S1 and S2 (Figures 2A,B). Several important adaptive mutations occur in HA, including E190D and G225D for H1N1 and Q226L and G228S for H2N2 and H3N2 (31). Some mutations in the polymerase subunits PB1, PB2, and PA are critical for increasing polymerase activity and viral virulence (32). As of 2 June 2020, more than ten thousand mutant sequences of SARS-COV-2 were uploaded to the GISAID database (13). There were 18,539 nonsynonymous mutations on the spike protein, the D614G mutation was unusually enriched and present in more than 8,000 strains. Up to February 1st 2021, nearly 300,000 strains containing D614G variation, A222V and L18F were the second and third mutations (Figure 2C). N439K, S477N and N501Y which located in the receptor-binding domain may affect the immunogenicity or vaccination. Our result showed S477N and N439K mutations have the capability to enhance the affinity with receptor (Figures 2D,E). Additional studies are needed to elucidate the effect of mutations in which are not located in the receptor-binding domain (pocket) on influenza virus and SARS-COV-2 infection and their epidemiological outcomes.

## Discussion

To date, most epidemiological reports have clarified the case disparities in clinical manifestations, routine blood tests, and immunity factors of COVID-19 (33–35). However, the dual epidemics of COVID-19 and influenza makes the diagnosis, treatment, and vaccination face greater challenges, even though some studies have assessed the differences between influenza and COVID-19 in terms of clinical characteristics or outcomes (36, 37). Both are respiratory virus infections, and influenza and COVID-19 have many of the same symptoms, including respiratory system and gastrointestinal system symptoms. Severe cases also have loss of taste or smell, difficulty breathing, or shortness of breath (38). COVID-19 may cause gastrointestinal problems, such as diarrhea, vomiting, and abdominal pain (39). Both the SARS-COV-2 receptor ACE2 and cellular serine protease transmembrane protease serine 2 (TMPRSS2) are critical for the fusion of viral and cellular membranes, which are not only expressed in lung alveolar type 2 cells and gland cells but also highly expressed in the ileum and colon (40, 41), suggesting that the virus can invade the digestive tract and intestine, and viral RNA can be detected in patients' stool.

Certain risk factors predispose patients to increased morbidity and mortality following exposure to the influenza A virus or SARS-COV-2. Age is the most significant risk factor for influenza and COVID-19-related mortality (42, 43). Age may also have different effects on the results of influenza virus infection between males and females (44). For COVID-19, the sex difference is a significant factor for mortality. Recently reported data showed that the male mortality rate is 2.4 times that of females (70.3 vs. 29.7%) (43), and there were 1641 men among the 2248 confirmed COVID-19 patients, which was 2.6 times the proportion of women (73 vs. 27%) (45). Chronic respiratory diseases and cardiovascular diseases are the two major comorbidities for influenza patients (5), and other comorbidities associated with poor influenza outcomes include diabetes and obesity. In our study, the most common underlying diseases in both flu and COVID-19 patients were hypertension, diabetes, and coronary heart disease. There was a significant difference in the progressive symptoms and complications of pneumonia between influenza and COVID-19 illness, including concurrency and CT radiology features. Only 17% of flu patients developed pneumonia, while the number increased to 90.67% in COVID-19 patients. In the early stage of flu pneumonia, X-ray imaging showed thickened and blurred lung texture and small patchy shadows. The advanced stage (onset 3-7 days) is dominated by GGOs and consolidation (46). COVID-19 pneumonia shows limited patchy shadows in the early stage, diffuse lung abnormalities, and multiple consolidations when the lesions are severe (47).

Dynamic changes in routine blood parameters refer to the evaluation of treatment or examination of the disease by observing the quantity change and shape distribution in blood cells. The total count of WBCs, neutrophils, lymphocytes, and monocytes progressively decreased, which may be related to the direct invasion of the virus into hematopoietic cells (48). The initial CRP of severe COVID-19 patients increased prior to CT findings (49), and the CRP value increased rapidly after admission, indicating a strong inflammatory response; the virus is prevalent in patients' bodies at this stage. ESR, elevated by the acute-phase response, can be used as an important indicator to distinguish patients with severe COVID-19 in the early stage (50). Severe COVID-19 illness is associated with a prominent elevation in ESR, which may provide additional information on disease progression. The BUN and blood creatinine levels rise rapidly before death in severe COVID-19 patients, this is consistent with our study (51). The inadequate sample size may limit the conclusion of our study, further studies are needed to analyze the dynamic changes in immune factors for COVID-19 progression and prognosis.

Several adaptation mutations have been identified in different segments of influenza A virus, and thoroughly researched mutations, including E627K and D701N on PB2 of avian flu virus and other subtypes, promote polymerase activity and adaption to act cooperatively with humans (52, 53). Adaptive mutations in SARS-COV-2 have also been reported, and the most important mutation in spike is D614G, which has attracted global attention (54, 55). The D614G mutant began to spread in Europe in early February 2020 and became the mainstream strain worldwide by May 2020, accounting for nearly 70% of samples in Europe and North America (56). Zhang et al. (57) latest research showed that small genetic mutations in the SARS-COV-2 variants are prevalent throughout Europe and the United States, which may increase the number of spike proteins on virions, and it will greatly improve the viral infectivity by 9-10 times. The N439K variants can maintain fitness but can evade the antibody-mediated immunity (58), it is unclear what the S477N mutation will bring to the vaccines and epidemics. More studies are needed to compare the clinical outcomes and prevalence between adaptive mutants and wild type.

This study provides new insight into the differences in clinical outcomes, laboratory abnormalities, comorbidities and hospitalization between influenza and COVID-19 patients and discusses the relationship between viral adaptive mutations and protein function, which will provide a reference for clinical differential diagnosis and epidemic surveillance.

## Data Availability Statement

The original contributions presented in the study are included in the article/supplementary material, further inquiries can be directed to the corresponding author/s.

## Ethics Statement

Written informed consent was obtained from the individual(s) for the publication of any potentially identifiable images or data included in this article.

## Author's Note

COVID-19 remains one of the key threats to public health. SARS-COV-2 causes a mild respiratory system disease, which has symptoms similar to those of seasonal influenza virus. COVID-19 and seasonal influenza both emerged in the respiratory virus season. Comparison of clinical features and outcomes of medically attended COVID-19 and influenza A patients will help us to better diagnose and treat the infection caused by SARS-COV-2. In this study, we systematically contrasted the proportions or values of epidemiologic characteristics, clinical features, blood abnormalities, progressive symptoms, and hospitalization between influenza and COVID-19. The parameters of coinfection patients who presented with both COVID-19 and flu were also compared with the COVID-19 cases. In addition, mutants of influenza virus HA and SARS-COV-2 spike causing the epidemic were resolved and discussed according to the literature or computation. This comparative research aims to aid pandemic control and be beneficial to clinical diagnosis.

## Author Contributions

LL, FZ, JY, and ZxL contributed to the design of experiments. LL, FZ, JR, SY, MJ, XuL, ZjL, XiL, WD, YL, and HT contributed to the conduction of experiments. LL, FZ, JR, SY, MJ, and ZjL contributed to the reagents. LL, FZ, JR, SY, MJ, XuL, ZjL, XiL, JL, JZ, and ZxL contributed to the analyses of the data. LL and ZxL contributed to the writing the paper. JY and ZxL contributed to the editing the paper. All authors contributed to the article and approved the submitted version.

## Conflict of Interest

The authors declare that the research was conducted in the absence of any commercial or financial relationships that could be construed as a potential conflict of interest.

## References

[1] Coronaviridae Study Group of the International Committee on Taxonomy of V. The species Severe acute respiratory syndrome-related coronavirus: classifying 2019-nCoV and naming it SARS-CoV-2. Nat Microbiol. (2020) 5:536–44. 10.1038/s41564-020-0695-z 32123347 PMC7095448

[2] Li Q Guan X Wu P Wang X Zhou L Tong Y. Early transmission dynamics in Wuhan, China, of novel coronavirus-infected pneumonia. N Engl J Med. (2020) 382:1199–207. 10.1056/NEJMoa2001316 31995857 PMC7121484

[3] University JH. COVID-19 Dashboard by the Center for Systems Science and Engineering (CSSE) at Johns Hopkins University. (2020). Available online at: https://www.arcgis.com/apps/opsdashboard/index.html#/bda7594740fd40299423467b48e9ecf6

[4] Dhama K Sharun K Tiwari R Dadar M Malik YS Singh KP. COVID-19, an emerging coronavirus infection: advances and prospects in designing and developing vaccines, immunotherapeutics, and therapeutics. Hum Vaccin Immunother. (2020) 16:1232–8. 10.1080/21645515.2020.1735227 32186952 PMC7103671

[5] Organization WH. Sex, Gender and Influenza. WHO (2010).

[6] Franco-Paredes C Hernandez-Ramos I Del Rio C Alexander KT Tapia-Conyer R Santos-Preciado JI. H1N1 influenza pandemics: comparing the events of 2009 in Mexico with those of 1976 and 1918-1919. Arch Med Res. (2009) 40:669–72. 10.1016/j.arcmed.2009.10.004 20304254

[7] Chuah CXP Lim RL Chen MIC. Investigating the Legacy of the 1918 influenza pandemic in age-related seroepidemiology and immune responses to subsequent influenza A(H1N1) viruses through a structural equation model. Am J Epidemiol. (2018) 187:2530–40. 10.1093/aje/kwy192 30165573 PMC6269251

[8] Kim H Webster RG Webby RJ. Influenza virus: dealing with a drifting and shifting pathogen. Viral Immunol. (2018) 31:174–83. 10.1089/vim.2017.0141 29373086

[9] Laha S Chakraborty J Das S Manna SK Biswas S Chatterjee R. Characterizations of SARS-CoV-2 mutational profile, spike protein stability and viral transmission. Infect Genet Evol. (2020) 85:104445. 10.1016/j.meegid.2020.104445 32615316 PMC7324922

[10] Lau SY Wang P Mok BW Zhang AJ Chu H Lee AC. Attenuated SARS-CoV-2 variants with deletions at the S1/S2 junction. Emerg Microbes Infect. (2020) 9:837–42. 10.1080/22221751.2020.1756700 32301390 PMC7241555

[11] Zhao JY Yan JY Qu JM. Interpretations of “Diagnosis and treatment protocol for novel coronavirus pneumonia (trial version 7)”. Chin Med J. (2020) 133:1347–9. 10.1097/CM9.0000000000000866 32301757 PMC7289291

[12] Health NHCOO. Influenza Diagnosis and Treatment Plan (2019 Version). (2019). Available online at: http://www.nhc.gov.cn/yzygj/s7653p/201911/a577415af4e5449cb30ecc6511e369c7/files/75a810713dc14dcd9e6db8b654bdef79.pdf

[13] GISAID. Global Initiative of Sharing All Influenza Data. (2020). Available online at: https://www.gisaid.org

[14] Group TSCG. COVID-19 Viral Genome Analysis Pipeline [Online]. (2020). Available online at: https://cov.lanl.gov/content/index

[15] Xue X Shi J Xu H. Dynamics of binding ability prediction between spike protein and human ACE2 reveals the adaptive strategy of SARS-CoV-2 in humans. Sci Rep. (2021) 11:3187. 10.1038/s41598-021-82938-2 33542420 PMC7862608

[16] Health NHCOO. DiagnosisandTreatmentProtocolforNovelCoronavirusPneumonia(6rdInterim Edition) [Online]. (2020). Available online at: http://www.gov.cn/zhengce/zhengceku/2020-02/19/content_5480948.htm

[17] Pal M Berhanu G Desalegn C Kandi V. Severe acute respiratory syndrome coronavirus-2 (SARS-CoV-2): an update. Cureus. (2020) 12:e7423. 10.7759/cureus.7423 32337143 PMC7182166

[18] Lu R Zhao X Li J Niu P Yang B Wu H. Genomic characterisation and epidemiology of 2019 novel coronavirus: implications for virus origins and receptor binding. Lancet. (2020) 395:565–74. 10.1016/S0140-6736(20)30251-8 32007145 PMC7159086

[19] Wang Q Zhang Y Wu L Niu S Song C Zhang Z. Structural and functional basis of SARS-CoV-2 entry by using human ACE2. Cell. (2020) 181:894–904.e899. 10.1016/j.cell.2020.03.045 32275855 PMC7144619

[20] Yan R Zhang Y Li Y Xia L Guo Y Zhou Q. Structural basis for the recognition of SARS-CoV-2 by full-length human ACE2. Science. (2020) 367:1444–8. 10.1126/science.abb2762 32132184 PMC7164635

[21] Zhou P Yang XL Wang XG Hu B Zhang L Zhang W. A pneumonia outbreak associated with a new coronavirus of probable bat origin. Nature. (2020) 579:270–3. 10.1038/s41586-020-2012-7 32015507 PMC7095418

[22] Coates BM Staricha KL Wiese KM Ridge KM. Influenza A virus infection, innate immunity, and childhood. JAMA Pediatr. (2015) 169:956–63. 10.1001/jamapediatrics.2015.1387 26237589 PMC4765914

[23] Rabaan AA Al-Ahmed SH Haque S Sah R Tiwari R Malik YS. SARS-CoV-2, SARS-CoV, and MERS-COV: a comparative overview. Infez Med. (2020) 28:174–84. 32275259

[24] Biggerstaff M Cauchemez S Reed C Gambhir M Finelli L. Estimates of the reproduction number for seasonal, pandemic, and zoonotic influenza: a systematic review of the literature. BMC Infect Dis. (2014) 14:480. 10.1186/1471-2334-14-480 25186370 PMC4169819

[25] Li R Pei S Chen B Song Y Zhang T Yang W. Substantial undocumented infection facilitates the rapid dissemination of novel coronavirus (SARS-CoV-2). Science. (2020) 368:489–93. 10.1126/science.abb3221 32179701 PMC7164387

[26] Petrosillo N Viceconte G Ergonul O Ippolito G Petersen E. COVID-19, SARS and MERS: are they closely related? Clin Microbiol Infect. (2020) 26:729–34. 10.1016/j.cmi.2020.03.026 32234451 PMC7176926

[27] Zhao Z Li H Wu X Zhong Y Zhang K Zhang YP. Moderate mutation rate in the SARS coronavirus genome and its implications. BMC Evol Biol. (2004) 4:21. 10.1186/1471-2148-4-21 15222897 PMC446188

[28] Jia Y Shen G Zhang Y Huang KS Ho Y Hor WS. Analysis of the mutation dynamics of SARS-CoV-2 reveals the spread history and emergence of RBD mutant with lower ACE2 binding affinity. bioRxiv. (2020). 10.1101/2020.04.09.034942

[29] Takahiko Koyama DP. Variant analysis of SARS-CoV-2 genomes. Bull World Health Organ. (2020) 98:495–504. 10.2471/BLT.20.253591 32742035 PMC7375210

[30] Nobusawa E Sato K. Comparison of the mutation rates of human influenza A and B viruses. J Virol. (2006) 80:3675–8. 10.1128/JVI.80.7.3675-3678.2006 16537638 PMC1440390

[31] Lazniewski M Dawson WK Szczepinska T Plewczynski D. The structural variability of the influenza A hemagglutinin receptor-binding site. Brief Funct Genomics. (2018) 17:415–27. 10.1093/bfgp/elx042 29253080 PMC6252403

[32] Taft AS Ozawa M Fitch A Depasse JV Halfmann PJ Hill-Batorski L. Identification of mammalian-adapting mutations in the polymerase complex of an avian H5N1 influenza virus. Nat Commun. (2015) 6:7491. 10.1038/ncomms8491 26082035 PMC4557292

[33] Puljiz I Kuzman I Dakovic-Rode O Schonwald N Mise B. Chlamydia pneumoniae and Mycoplasma pneumoniae pneumonia: comparison of clinical, epidemiological characteristics and laboratory profiles. Epidemiol Infect. (2006) 134:548–55. 10.1017/S0950268805005522 16316495 PMC2870427

[34] Guan WJ Ni ZY Hu Y Liang WH Ou CQ He JX. Clinical characteristics of coronavirus disease 2019 in China. N Engl J Med. (2020) 382:1708–20. 10.1056/NEJMoa2002032 32109013 PMC7092819

[35] Xu XW Wu XX Jiang XG Xu KJ Ying LJ Ma CL. Clinical findings in a group of patients infected with the 2019 novel coronavirus (SARS-Cov-2) outside of Wuhan, China: retrospective case series. BMJ. (2020) 368:m606. 10.1136/bmj.m606 32075786 PMC7224340

[36] Song X Delaney M Shah RK Campos JM Wessel DL DeBiasi RL. Comparison of clinical features of COVID-19 vs seasonal influenza A and B in US children. JAMA Netw Open. (2020) 3:e2020495. 10.1001/jamanetworkopen.2020.20495 32897374 PMC7489826

[37] Liu M Zeng W Wen Y Zheng Y Lv F Xiao. K. COVID-19 pneumonia: CT findings of 122 patients and differentiation from influenza pneumonia. Eur Radiol. (2020) 30:5463–9. 10.1007/s00330-020-06928-0 32399710 PMC7216854

[38] Prevention CfDCA. Similarities and Differences between Flu and COVID-19. (2020). Available online at: https://www.cdc.gov/flu/symptoms/flu-vs-covid19.htm

[39] Ng SC Tilg H. COVID-19 and the gastrointestinal tract: more than meets the eye. Gut. (2020) 69:973–4. 10.1136/gutjnl-2020-321195 32273292 PMC7211058

[40] Bilinska K Jakubowska P Von Bartheld CS Butowt R. Expression of the SARS-CoV-2 entry proteins, ACE2 and TMPRSS2, in cells of the olfactory epithelium: identification of cell types and trends with age. ACS Chem Neurosci. (2020) 11:1555–62. 10.1021/acschemneuro.0c00210 32379417 PMC7241737

[41] Lukassen S Chua RL Trefzer T Kahn NC Schneider MA Muley T. SARS-CoV-2 receptor ACE2 and TMPRSS2 are primarily expressed in bronchial transient secretory cells. EMBO J. (2020) 39:e105114. 10.15252/embj.20105114 32246845 PMC7232010

[42] Thompson WW Shay DK Weintraub E Brammer L Cox N Anderson LJ. Mortality associated with influenza and respiratory syncytial virus in the United States. JAMA. (2003) 289:179–86. 10.1001/jama.289.2.179 12517228

[43] Jin JM Bai P He W Wu F Liu XF Han DM. Gender differences in patients with COVID-19: focus on severity and mortality. Front Public Health. (2020) 8:152. 10.3389/fpubh.2020.00152 32411652 PMC7201103

[44] Thomas MR Marston L Rafferty GF Calvert S Marlow N Peacock JL. Respiratory function of very prematurely born infants at follow up: influence of sex. Arch Dis Child Fetal Neonatal Ed. (2006) 91:F197–201. 10.1136/adc.2005.081927 16418306 PMC2672701

[45] ICNARC. ICNARC Report on COVID-19 in Critical Care. (2020). Available online at: https://www.icnarc.org/About/Latest-News/2020/04/04/Report-On-2249-Patients-Critically-Ill-With-Covid-19

[46] Wu J Lu AD Zhang LP Zuo YX Jia YP. Zhonghua xue ye xue za zhi =. Zhonghua Xueyexue Zazhi. (2019) 40:52–7. 10.3760/cma.j.issn.0253-2727.2019.01.010 30704229 PMC7351698

[47] Committee CotIDRGoC.S.o.R., Infectious, o.t.I.D.R.S.o.C.M.D.A., Disease Imaging Group IDB, Chinese Research Hospital Association; Imaging,; Infectious Diseases Group, et al. (Infectious, C.o.C.A.f.t.P.a.T.o.S.A.I., Disease) Guideline for imaging diagnosis of novel coronavirus (2019-ncov) infected pneumonia (1st edition 2020). New Med. (2020) 30:22–34. 10.12173/j.issn.1004-5511.2020.01.07

[48] Lu G Wang J. Dynamic changes in routine blood parameters of a severe COVID-19 case. Clin Chim Acta. (2020) 508:98–102. 10.1016/j.cca.2020.04.034 32405079 PMC7217800

[49] Tan C Huang Y Shi F Tan K Ma Q Chen Y. C-reactive protein correlates with computed tomographic findings and predicts severe COVID-19 early. J Med Virol. (2020) 92:856–62. 10.1002/jmv.25871 32281668 PMC7262341

[50] Lapic I Rogic D Plebani M. Erythrocyte sedimentation rate is associated with severe coronavirus disease 2019 (COVID-19): a pooled analysis. Clin Chem Lab Med. (2020) 58:1146–8. 10.1515/cclm-2020-0620 32386190

[51] Wang D Hu B Hu C Zhu F Liu X Zhang J. Clinical characteristics of 138 hospitalized patients with 2019 novel coronavirus–infected pneumonia in Wuhan, China. JAMA. (2020) 323:1061–9. 10.1001/jama.2020.1585 32031570 PMC7042881

[52] Arai Y Kawashita N Hotta K Hoang PVM Nguyen HLK Nguyen TC. Multiple polymerase gene mutations for human adaptation occurring in Asian H5N1 influenza virus clinical isolates. Sci Rep. (2018) 8:13066. 10.1038/s41598-018-31397-3 30166556 PMC6117316

[53] Venkatesh D Bianco C Nunez A Collins R Thorpe D Reid SM. Detection of H3N8 influenza A virus with multiple mammalian-adaptive mutations in a rescued Grey seal (Halichoerus grypus) pup. Virus Evol. (2020) 6:veaa016. 10.1093/ve/veaa016 32211197 PMC7079721

[54] Daniloski Z Jordan TX Ilmain JK Guo X Bhabha G tenOever BR. The Spike D614G mutation increases SARS-CoV-2 infection of multiple human cell types. bioRxiv. (2020). 10.1101/2020.06.14.151357 33570490 PMC7891930

[55] Eaaswarkhanth M Al Madhoun A Al-Mulla F. Could the D614G substitution in the SARS-CoV-2 spike (S) protein be associated with higher COVID-19 mortality? Int J Infect Dis. (2020) 96:459–60. 10.1016/j.ijid.2020.05.071 32464271 PMC7247990

[56] Hu J He CL Gao QZ Zhang J Cao XX Long QX. The D614G mutation of SARS-CoV-2 spike protein enhances viral infectivity and decreases neutralization sensitivity to individual convalescent sera. bioRxiv. (2020). 10.1101/2020.06.20.161323

[57] Zhang L Jackson CB Mou H Ojha A Rangarajan ES Izard T. The D614G mutation in the SARS-CoV-2 spike protein reduces S1 shedding and increases infectivity. bioRxiv. (2020). 10.1101/2020.06.12.148726 33243994 PMC7693302

[58] Emma CT Laura ER James GS Roberto S Ana SF Jason AW. Circulating SARS-CoV-2 spike N439K variants maintain fitness while evading antibody-mediated immunity. Cell. (2021). 10.1016/j.cell.2021.01.037 33621484 PMC7843029

